# Prebiotic activity of garlic (*Allium sativum*) extract on *Lactobacillus acidophilus*

**DOI:** 10.14202/vetworld.2019.2046-2051

**Published:** 2019-12-24

**Authors:** Prayogi Sunu, Dwi Sunarti, Luthfi Djauhari Mahfudz, Vitus Dwi Yunianto

**Affiliations:** 1 Department of Animal Science, Faculty of Animal and Agricultural Sciences, Diponegoro University, Semarang, Indonesia; 2 Department of Animal Science, Faculty of Animal Science, Boyolali University, Boyolali, Indonesia

**Keywords:** *Allium sativum*, prebiotics, probiotics, synbiotics

## Abstract

**Aim:**

The study aimed to examine the ability of prebiotic concentrations to increase the growth of probiotic bacteria *in vitro*.

**Materials and Methods:**

The probiotics used were *Lactobacillus acidophilus* and garlic (*Allium sativum*) extract.

**Results:**

The results showed that garlic can increase the growth of *L. acidophilus* bacteria with the lowest concentration of 4% being the most effective (p<0.05). Increased fructooligosaccharide (FOS) content in garlic can increase the significant growth of *L. acidophilus* as a probiotic bacterium.

**Conclusion:**

The results showed that garlic can increase the growth of *L. acidophilus* bacteria by a minimum of 4% (p<0.05). Adding FOS to garlic can increase the significant growth of *L. acidophilus* as a probiotic bacterium.

## Introduction

Antibiotics have been used in the broiler farm industry for decades. Regarding the issue of food security, antibiotic growth promoters (AGPs) are used in most countries [1]. This is related to the potential of AGPs to cause resistance in humans as consumers [2]. Because the ban on AGPs can have a negative impact on the health and productivity of chickens, an alternative substitute for AGPs is needed.

Prebiotics are substrates or undigested food [3] that are selectively fermented by several microflora that live in the digestive tract, such as *Lactobacillus* and *Bifidobacterium*, which has a beneficial effect on health [3]. Prebiotics function to stimulate the growth and activity of bacteria that have beneficial effects on the health of the host, especially non-pathogenic bacteria. Gilchrist *et al*. [1] reported that almost every oligo- and polysaccharide is a prebiotic, but not all carbohydrate foods are prebiotic. According to Kareem *et al*. [4], there are at least three criteria that need to be fulfilled by a material so that it categorized as prebiotic; (1) it cannot be hydrolyzed nor absorbed at the top of the gastrointestinal tract so that it can reach the colon without structural changes and not be excreted in the feces [5]; (2) it must be selected and fermented by a number of beneficial microflora in the colon to produce a beneficial effect on the host and stimulate the growth of bacteria that actively carry out metabolism [6]; (3) it must be able to convert the colon microflora into compositions that benefit health and selectively stimulate the growth activity of bacteria, such as *Lactobacillus* in the colon [7].

Allicin and other sulfur components in garlic are active ingredients with antibacterial effects [8]. Garlic has an antibacterial activity that is quite effective at fighting various kinds of Gram-negative and/or positive bacteria [9]. Some bacteria that have been shown to have a high sensitivity to the antibacterial activity of garlic are *Staphylococcus*, *Mycobacteria*, and *Proteus* species [10]. Studies have reported that garlic can be used as a natural prebiotic in feed at a level of 1.0% to improve growth performance [11].

However, studies on the use of garlic as a synbiotic with *Lactobacillus acidophilus* are still limited. This research aimed to examine the effect of optimal prebiotic concentration in increasing the growth of probiotic bacteria *in vitro*.

## Materials and Methods

### Ethical approval

This study has been approved by the Animal Ethics Committee of the Faculty of Animal and Agricultural Sciences, Diponegoro University, Semarang, Indonesia.

### Garlic extract

The prepared garlic material (3 kg) was washed and peeled. After peeling, the garlic was washed again, sliced to a thickness of 1 cm, and placed into a juicer to obtain the extract. The obtained extract was then filtered. After the screening process, the extract was sterilized by pasteurization for 10 min. The extract was stored in a clean container in a dark place at a temperature below 20°C. The amount of extract obtained was recorded and compared to the amount of raw material used.

### Synbiotics

Garlic was extracted at different concentrations (2 ml, 4 ml, 6 ml, and 8 ml), then added to *L. acidophilus* at the ratio of 100:1 (ml). Isolates from de Man, Rogosa, and Sharpe (MRS) media supplemented with 20% glycerol (v/v) and rejuvenated bacterial culture on MRS media were incubated at 37°C for 24-48 h to activate the bacteria, which were then inoculated into an MRS broth media as much as 1 oose and incubated at 37°C for 24 h [12].

### Testing of L. acidophilus against bile acids

*L. acidophilus* was grown anaerobically in a solution of MRS broth at a temperature of 37°C. Then, 24 h old liquid stock culture of *L. acidophilus* was inoculated into MRS broth at a ratio of 2:20 (ml) and incubated at 30°C for 18 h. The same procedure was performed to test the bacterial pH, with pH 1.5-6.5 [13]. One milliliter of culture was taken and placed in 9 ml of MRS broth, the pH was set to 1.5-6.5 with 1 N HCl and incubated at 30°C for 24 h. The number of colonies grown was observed by the planting technique on pour plates containing MRS agar (qualitative analysis).

### Testing of L. acidophilus against bile salts

Tests were carried out using the method, namely, by preparing 1 ml of lactic acid bacteria (LAB) in 20 h old MRS broth added to 9 ml of 0.65% NaCl. After checking serially, a plate count with the pouring method was performed on MRS agar containing 500 ppm and 1000 ppm Oxygall (Oxoid)/bile salts, with MRS agar as a control. All test mixtures were incubated at 37°C. Resistance to bile salts was calculated based on the difference in the unit log number of colonies growing in control conditions with bile salt treatments. Observations were made by comparing the number of *L. acidophilus*: The smaller the difference, the more resistant the tested strains were against bile salts [14].

### Viability test of L. acidophilus in garlic mixture

The resistance of *L. acidophilus* in the mixture was calculated using the cup count method [15], 4 h after mixing. The sample was prepared and homogenized, and 1 ml of each dilution was pipetted to a Petri dish of 12-15 ml. The samples were homogenized by carefully shaking the Petri dishes until they were evenly mixed for seeding. A blank examination was carried out by mixing seeded diluted water for each sample examined and then the mixtures were left to freeze. The Petri dishes are inserted into the incubator and incubated at 35±10°C for 24-48 h. The growth of milky white colonies on each cup contained 25-250 colonies after 48 h. The total plate number of *L. acidophilus* in 1 g or 1 ml is calculated by dividing the average number of colonies in the cup with the dilution factor used (accordingly).

### Calculation of total LAB in feed

The calculation of total bacteria was performed by taking a sample of 1 g and then adding as much as 9 ml of 0.9% NaCl solution until homogeneous. Then, the food suspension was placed in a Petri dish and wrapped in aluminum foil and stored for 24 h. The calculation of the number of colonies was performed using a colony counter.

### Temperature resistance test

One colony of bacterial isolates was inoculated into MRS broth media with a mixture of garlic and basal feed and then incubated at a temperature of 50, 60, 70, or 80°C. The bacterial growth was determined by calculating bacterial colonies with dilution techniques and characterized by the turbidity in the media.

### Old feed storage test

The homogeneous ingredients were evenly sprayed into 100 g of feed using a spray bottle, dried for 15 min, stored in a closed container (jar) at room temperature (25°C), and (according to the treatment) stored for 1, 2, 3, or 4 days. The numbers of colonies were calculated using a colony counter.

### Statistical analysis

Analysis of the data obtained was performed using Steel and Torrie analysis of variance (ANOVA) if there were differences between treatments as determined by Duncan’s multiple range test [16]. The linear model that explains each value of observation using a model proposed by Gherezgihier *et al*. [16] was as follows:

*Y_ij_* = *µ* + *τ_i_* + *ε_ij_*.

Information:

*Y_ij_* = Observation of the second treatment effect and test *j*;

*μ* = General average value;

*τ_i_* = Effect of treatment *i*;

*ε_ij_* = Test error due to maintenance and test *j*.

Hypothesis statistics tested:

H_0_: *τ*_1_ = *τ*_2_ = *τ*_3_ = 0;

There was no effect on the treatment of the growth test of *L. acidophilus* bacteria on liquid medium.

H_1_: There is at least one *τ* ≠ 0;

There is an effect of the treatment of the growth test of *L. acidophilus* bacteria on liquid medium.

The data obtained were tested using ANOVA at 5% level. If there was a real effect and treatment H_1_ was accepted, then it was followed by Duncan’s multiple range test [16]. The test criteria used for statistical analysis were as follows:

*F* count < *F* table (0.05), H_0_ accepted and H_1_ rejected;

*F* count ≥ *F* table (0.05), H_0_ rejected and H_1_ accepted.

## Results and Discussion

The results of the study of fructooligosaccharide (FOS) content in garlic and the effect of FOS from garlic on the growth of *L. acidophilus* bacteria are presented in Tables-1 and 2.

**Table-1:** Results of testing for FOS content in garlic.

Test parameters	Results	Unit	Method
FOS	3.34	% b/b	HPCL

FOS=Fructooligosaccharide

**Table-2:** Potential of garlic as a prebiotic against bile acids, bile salts, temperature, and feed (log CFU/ml).

Treatment	Synbiotic garlic				Control

	A (2 ml)	B (4 ml)	C (6 ml)	D (8 ml)	
Bile salt	9.97^b,c^	10.42^a^	10.08^b^	9.83^c,d^	9.78^c,d^
Bile acid	8.76^b^	8.96^a^	8.76^b^	8.74^b^	8.74^b^
Feed	8.93^b^	9.38^a^	8.91^b^	8.81^b^	8.86^b^

a,b,c,d Superscripts in the column show significant (p<0.05)

The results of this study (Table-1) indicate that the FOS content in garlic was 3.34% b/b. This is consistent with the study of Altuntas and Korukluoglu [17], which reported that FOS is preferred and fermented by *Lactobacillus* and *Bifidobacterium*. FOS and galactooligosaccharides are a blend of the composition of oligosaccharides (medium-chain carbohydrates) [18]. FOS compounds are found in fruits and vegetables, including onion tubers, such as garlic (1-2%) [19].

**Figure-1:**
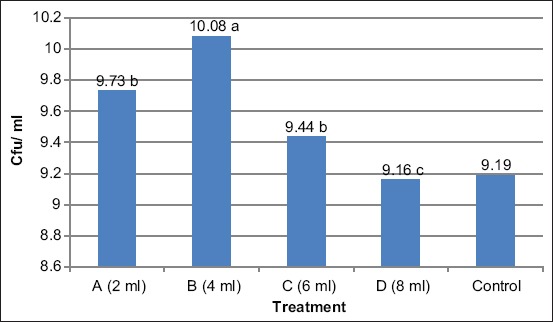
Optimal level of garlic. ^a,b^Superscripts in the line show significant (p<0.05). A=Extract of 2 ml of garlic, B=Extract of 4 ml of garlic, C=Extract of 6 ml of garlic, D=Extract of 8 ml of garlic.

The results of this study (Figure-1) show that the optimal level of garlic in sample B (4 ml) has a very significant effect (p<0.05) compared to that of the control, A, C, and D samples. This is consistent with Lopes *et al*. [20], who reported that with the addition of foods containing prebiotics such as oligosaccharides, the composition of *Lactobacillus* spp., increased by 81%. Supplementation with prebiotics at 0.1-0.2% can increase beneficial bacteria and reduce detrimental populations [12].

### Acidity

This study (Table-2) shows that the growth of *L. acidophilus* with pH 2 and pH 4 in sample B (4 ml of synbiotic) had a very significant effect (p<0.05) compared to the control, A (2 ml), C (6 ml), and D (8 ml) samples, so it can be said that *Lactobacillus* is resistant to acid and able to survive in pH 2 low pH conditions. According to Gupta and Sharma [21], enzymes affect the growth of LAB at low pH. The enzymes that affect the resistance of LAB at low pH levels are protease enzymes. Furthermore, it is said that the higher the protease enzyme possessed by an isolate, the more it can increase its resistance to acidic conditions. One of the protease enzymes, aminopeptidase, can affect the adaptation and growth of LAB isolates under acidic conditions [22]. This situation is not beneficial for pathogenic microorganisms. Damayanti *et al*. [23] argued that LAB can survive acid damage due to the presence of histidine decarboxylase enzymes and deimination of arginine enzymes. These results indicate that only a few isolates of the LAB tested can pass through the acidic gastric tract. The tolerance of LAB to acid is quite high due to its ability to maintain cytoplasmic pH at a higher alkalinity than the extracellular pH [13]. This is because if probiotics enter the digestive tract, they must survive the acidic conditions of the stomach, where the pH is around 2 [24]. According to Jiang *et al*. [25], the HCl concentration is 0.2-0.5%, reducing the gastric pH to 1 if it is completely empty. LAB resistance to pH is low due to their ability to maintain an internal pH more alkaline than the external pH and by having cell membranes that are more resistant to cell leakage due to low pH [26]. The sensitivity of bacteria to acids can depend on the simultaneous work of other additional factors, such as water activity, salinity, redox potential, and heat treatment [27].

### Bile salts

The observation (Table-2) shows that *L. acidophilus* cells can grow and survive in bile salts with a concentration of 0.3%, and 0.6% in sample B (4 ml of synbiotic) has a very significant effect (p<0.05) compared to that of the control, A (2 ml), C (6 ml), and D (8 ml) samples. To see the resistance of LAB to bile salts can be seen from the number of log differences in the number of bacterial colonies, which, according to Vecchione *et al*. [28], observations of resistance to bile salts were calculated based on the difference in the unit log number of colonies grown in control conditions with bile salt treatments. The smaller the resistance, the more strains are tested against bile salts. The results of the study are in accordance with those of O’Flaherty *et al*. [29], which suggested that probiotics are living microorganisms that enter in sufficient quantities so that they can provide health benefits for the host. The sufficient amount stated by the Food and Agriculture Organization of the United Nations and the World Health Organization is 10^6^-10^8^ CFU/g. Live probiotics must be able to survive in unfavorable conditions, such as exposure to stomach acid and bile salt, and maintain their physiological activities [30].

### Total growth of LAB in feed

The results of the study (Table-2) showed that the addition of garlic can significantly increase (p<0.05) the growth of *L. acidophilus* LAB on artificial ration feed, namely, in sample B (4 ml synbiotic). This is in accordance with the study of Georgieva *et al*. [31], which found that that the viability (resistance) of *L. acidophilus* in factory chicken feed can survive so that chicken feed containing these microorganisms is used. Because chicken feed containing microorganisms has a lifespan or a time limit till expiration, which is characterized by the death of microorganisms in chicken feed. If the microorganisms in the feed are dead, then the feed is no longer able to inhibit pathogenic bacteria. According to Javanmard *et al*. [32], the growth and survival of organisms are reduced due to the limited food or nutritional supply. Under limited food conditions, the number of organisms will decline quickly. Indeed, cell death is greater than cell proliferation [33]. This is in accordance with Paulina Markowiak and Śliżewska [34], who reported that the declining growth rate occurs because mortality increases, reproduction decreases, or both occur. According to Tokatl *et al*. [35], this situation shows that there is competition among bacteria for nutrition and space. It is also caused by the presence of cells that are added to the medium. According to Lee *et al*. [18], in some circumstances, it can be followed by the lysis of cells so that the turbidity and number of cells directly calculated will decrease in line with the reduction of living cells. There is further inhibition of metabolite products and important nutrients in the depleted medium.

### The amount of *L. acidophilus* bacteria at different temperatures

Data in this study (Table-3) showed that *L. acidophilus* had the highest amount of growth in sample B, which had a very significant effect (p<0.05) with the control, C, and D samples, but was not significantly different from sample A at different temperatures. This is in accordance with Schloss [36], who reported that the higher the temperature given, the smaller the activity or possibility of live bacteria. This is because the temperature can affect microorganisms in two opposite ways: (1) When the temperature rises, the speed of metabolism rises and growth is accelerated. Conversely, when the temperature drops, the speed of metabolism also decreases and growth is slowed. (2) If the temperature rises or falls, the growth rate may stop, the cell components become inactive, and cells can die. According to Fernández *et al*. [2], this occurs because lactic acid, as a short-chain fatty acid, is also used as a carbon source by *L. acidophilus* for its growth through metabolic pathway β-oxidation such that lactic acid metabolism is directly used as a source of nutrition by *L. acidophilus*, thereby increasing bacterial growth.

**Table-3:** The amount of *Lactobacillus acidophilus* bacteria at different temperatures.

Treatment	Temperature resistance			

	50°C	60°C	70°C	80°C
A (2 ml)	7.92^a^	7.94^a^	7.87^a,b^	8.01^a,b^
B (4 ml)	8.00^a^	8.10^a^	8.00^a^	8.19^a^
C (6 ml)	7.77^b^	7.76^b^	7.74^b^	7.82^b^
D (8 ml)	7.77^b^	7.75^b^	7.74^b^	7.86^b^
Control	7.75^b^	7.76^b^	7.75^b^	7.80^b^

a,b Superscripts in the column show significant (p<0.05)

### The amount of *L. acidophilus* bacteria against storage time

Based on Table-4, it is known that the storage time that feeds could maintain the amount of bacteria in sample B was significantly different (p<0.05) than that in the control, C, and D samples, but not significantly different from sample A at 1, 2, 3, and 4 days storage periods. This is presumably because the LAB population increases with the length of storage time, provided that bacteria get nutritional support to maintain their metabolism and are provided with the right conditions for growing bacteria; *Lactobacillus*, for example, live at optimum temperatures of 30°C under anaerobic conditions (no need for O_2_ free) [37]. Table-4 shows that the population of LAB increases in population along with the storage time of the biotic feed and temperature; the optimal temperature for bacterial growth is room temperature above 50°C. According to Amalia *et al*. [38], the time between each microbial cell division ranges from 10 to 60 min. According to Yang *et al*. [39], the optimum temperature for the growth of LAB varies in each strain. The LAB *Bacillus subtilis* belongs to the class of mesophilic bacteria whose optimum temperature is 25°C and maximum temperature is 40°C.

**Table-4:** The amount of *Lactobacillus acidophilus* bacteria against storage time.

Treatment	Storage time			

	1 day	2 days	3 days	4 days
A (2 ml)	7.92^a,b^	7.88^a,b^	7.91^a,b^	8.05^a^
B (4 ml)	7.98^a^	8.00^a^	8.05^a^	8.25^a^
C (6 ml)	7.80^b^	7.76^b^	7.78^b^	7.78^b^
D (8 ml)	7.78^b^	7.83^b^	7.84^b^	7.82^b^
Control	7.77^b^	7.76^b^	7.77^b^	7.77^b^

a,b Superscripts in the column show significant (p<0.05)

## Conclusion

For the addition of garlic as a prebiotic with *L. acidophilus*, which can be used as a synbiotic, 4 ml of extract is the most effective at increasing the growth of *L. acidophilus*, acid and bile salt resistance, feed viability, temperature resistance, and feed storage time.

## Authors’ Contributions

PS performed research to completion, data analysis, and scientific article writing. DS, LDM, and VDY performed data analysis, drafted and revised the manuscript. All authors read and approved the final manuscript.
